# Functional characterization of DPP4 and FcRn as receptor and coreceptor for classical human astroviruses in Caco-2 cells

**DOI:** 10.1371/journal.ppat.1013316

**Published:** 2025-07-18

**Authors:** Catalina Aguilera-Flores, María del Pilar Valencia-Morales, Tomás López, Joaquín Moreno-Contreras, Adam Lentz, Marco A. Espinoza, Rebecca M. DuBois, Susana López, Carlos F. Arias

**Affiliations:** 1 Departamento de Genética del Desarrollo y Fisiología Molecular, Instituto de Biotecnología, Universidad Nacional Autónoma de México, Cuernavaca, Morelos, Mexico; 2 Department of Biomolecular Engineering, University of California, Santa Cruz, California, United States of America; US Food and Drug Administration, UNITED STATES OF AMERICA

## Abstract

Classical human astroviruses (HAstV) are a global cause of viral gastroenteritis, particularly in children and immunocompromised individuals. Despite their clinical significance, the biology of HAstV remains poorly understood. In particular, the identification of cellular receptors and coreceptors has been elusive. Recent studies have identified the human neonatal Fc receptor (FcRn) as a functional receptor and dipeptidyl peptidase IV (DPP4) as an entry factor for HAstV. However, the precise roles of FcRn and DPP4 during HAstV infection are unknown. To learn about their function, we used FcRn-knockout (KO), DPP4-KO, and FcRn/DPP4 double-KO Caco-2 cells generated via CRISPR/Cas9. Our results showed that DPP4 serves as the receptor for classical HAstV. In contrast, infectious virus assays and confocal fluorescence microscopy revealed that FcRn acts as a coreceptor, facilitating viral internalization and the release of the RNA genome. The half-time for HAstV-1 genome uncoating was delayed threefold in FcRn-KO Caco-2 cells compared to WT cells. Additionally, the characterization of HAstV-8 variants with reduced FcRn binding capacity allowed the identification of two amino acids in the viral capsid spike protein, D471 and N512, critical for the spike-FcRn interaction. These amino acid residues are part of the epitope footprint of neutralizing monoclonal antibodies (Nt-MAbs) to HAstV previously mapped by X-ray crystallography. Further experiments using virus infectivity and attachment assays, along with Nt-MAbs targeting HAstV-1, suggest that the binding sites for FcRn and DPP4 are spatially proximal on the viral spike, defining a functional domain for cell infection. Notably, the infectivity of the divergent HAstV-VA1 was independent of these two proteins, highlighting the receptor variability across HAstV clades. These findings provide new insights into the mechanism of HAstV infection, offering relevant implications for the development of antiviral therapies and vaccines targeting this significant human pathogen.

## Author summary

Classical human astroviruses (HAstV) are a significant component of the global burden of diarrheal diseases in childhood, yet their biology remains poorly understood. In this report, we have identified DPP4 as the receptor mediating the initial binding of the virus to the cell surface, and FcRn as a coreceptor, facilitating clathrin-mediated endocytosis of the virus and the release of viral genomic RNA into the cytoplasm. We used mutant viral strains and knockout cells to show that DPP4 and FcRn interact at spatially proximal binding sites on the virus spike protein, overlapping with critical regions recognized by neutralizing antibodies. We propose a two-step HAstV cell entry mechanism with distinct roles for DPP4 and FcRn. In contrast, the divergent HAstV strain (HAstV-VA1) does not depend on these cellular molecules for infection. These insights into HAstV entry mechanisms contribute to understanding its pathogenesis and inform the development of antivirals and vaccines.

## Introduction

Human astroviruses (HAstV) are a significant cause of viral gastroenteritis worldwide, particularly affecting vulnerable populations such as children, older people, and immunocompromised individuals [1,2]. First identified in 1975, HAstV has since been recognized as a significant cause of the global burden of diarrheal diseases [3]. HAstV are classified into three clades: one comprising classical HAstV (serotypes 1–8), with serotype 1 being the most prevalent globally [2], and two more clades formed by the more recently described genetically distinct HAstV clades VA and MLB. The association of these divergent viruses with human gastroenteritis has not been established [4,5]. Still, they have been related to central nervous system diseases in immunocompromised hosts, including encephalitis and meningitis [6]. Despite the increasing recognition of HAstV as a diverse family of viruses with broad clinical manifestations, the molecular mechanisms underlying their entry into host cells are poorly understood.

HAstV are small (ca. 35 nm in diameter), nonenveloped viruses with a single-stranded, positive-sense RNA genome of about 6.8 kb that comprises four open reading frames (ORFs) [1,7,8]. ORF1a and ORF1b encode the nonstructural proteins of the virus, while ORF2 encodes the capsid protein precursor [1] and ORFX, which encodes a viroporin required at late stages of infection [8]. In the case of mature classical HAstV, the infectious virions are composed of two polypeptides generated by trypsin cleavage of the capsid protein precursor VP70 [9,10]. VP34 constitutes the shell of the virus particle, while VP27 forms the 30 dimeric globular spikes that protrude from the virion [7,9,10]. Trypsin treatment increases the infectivity of classical HAstV several log-fold compared to the untreated virions [10].

The mature virus enters the cell by clathrin-mediated endocytosis, requires endosome acidification, actin filament polymerization, and the presence of cholesterol in the cell membrane. The virus reaches late endosomes before releasing its RNA genome into the cell cytoplasm [11]; virus genome uncoating is facilitated by the host protein disulfide isomerase 4 (PDI4) [9]. However, the identity of the cellular receptors and coreceptors for HAstV is just emerging. Based on the crystal structure of the virus spike, glycans have been proposed as potential attachment factors for HAstV [12], but none have yet been identified to serve this role.

Recently, the neonatal Fc receptor (FcRn) was identified as a receptor for classical HAstV [13,14], and the dipeptidyl peptidase-4 (DPP4) as an entry factor for infection [14]. However, the detailed function of these molecules in HAstV infection has not been characterized. FcRn is an important component of immune regulation, facilitating the recycling of IgG and the transport of immunoglobulins across epithelial barriers [15]. DPP4 is a multifunctional type II transmembrane serine exopeptidase that contributes to the regulation of various physiological processes, including blood sugar homeostasis by cleaving peptide hormones, chemokines, and neuropeptides [16]. FcRn was previously described as a receptor for echoviruses [17–19] and arteriviruses [20,21], while DPP4 functions as a receptor for some human and animal coronaviruses, including MERS [22–24].

In this work, we elucidated the functional roles of FcRn and DPP4 for classical HAstV. To avoid confusion, throughout this paper we refer to receptor as the cell molecule that is essential for the virus to bind to the cell surface and leads to a productive infection, and coreceptor as the cell molecule that interacts with the virus after cell attachment and favors the internalization, and in some cases the uncoating of the virus particle. However, it is essential to note that the roles of the receptor and coreceptor can vary across different cell types, even for the same virus. We found that in Caco-2 cells, DPP4 functions as the virus receptor, while FcRn serves as a coreceptor, facilitating the internalization of infectious virions and probably genome uncoating. We also report that the interaction sites of FcRn and DPP4 lie spatially close on the surface of the virus spike and overlap with amino acid residues recognized by neutralizing antibodies to HAstV, representing a functional domain in the virus capsid spike. These findings advance our understanding of the molecular mechanisms driving viral entry and pave the way to a better understanding of host specificity. Furthermore, elucidating the roles of FcRn and DPP4 in cell entry expands our knowledge of HAstV pathogenesis and has implications for antiviral strategies and vaccine development.

## Results

### FcRn and DPP4 explain most of the classical HAstV infectivity in Caco-2 cells

**Fig 1:**
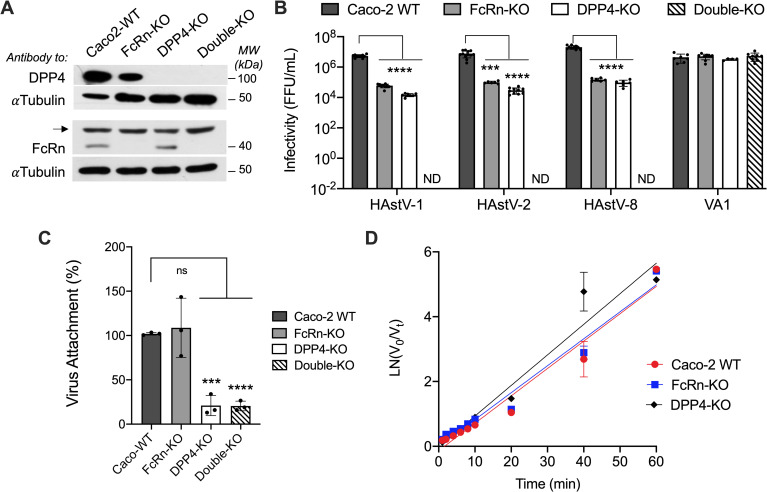
Infectivity and binding of HAstV in wild-type, FcRn-KO, and DPP4-KO Caco-2 cells. A) Western blot showing the loss of expression of the FcRn receptor in FcRn-knockout (FcRn-KO), DPP4 in DPP4-KO cells, and of both cellular molecules in the double-KO Caco-2 cells. The arrow points to a nonspecific band revealed with the FcRn antibody. Tubulin was used as a loading control in the gel. B) Infectivity of HAst-V serotypes 1, 2, and 8, and of the divergent HAstV-VA1 in WT and three knockout Caco-2 cell lines. The infectious virus titers of HAstV-infected cell lysates were determined in Caco-2 cell monolayers grown in 96-well plates. C) Purified HAstV-1 binding in WT and the three knockout Caco-2 cell lines. Purified virus (160 ng) was added for 1 h at 4°C to allow the virus to attach to the cell surface. After removing the unbound virus and washing the cells, the attached virus was detected by RT-qPCR, as described in the Materials and Methods section. Experiments were performed on ice to prevent virus endocytosis. The infectivity and binding assays were performed in three independent experiments, each with technical duplicates. Data are expressed as focus-forming units (ffu)/ml (B) or as percentages of the attachment to WT cells, determined by RT-qPCR (C), and represent the mean ± SD. Statistical significance was evaluated by a t-test using GraphPad Prism 9.0. *, ≤ 0.05; **, ≤ 0.01; ***, ≤ 0.001; ****, ≤ 0.0001. D) Infectious HAstV-1 binds with similar kinetics to WT, FcRn-KO, and DPP4 Caco-2 cells. Trypsin-treated HAstV-1 (at an MOI of 0.02) was added to the three Caco-2 cell lines and incubated at 4°C for the indicated times to allow virus attachment but not entry. The unbound virus was removed, the cells were washed twice, and the infection was allowed to proceed for 18 h at 37°C. Cells were then fixed and immunostained as described in the Materials and Methods section. The input virus (V_0_) was the infectious virus detected in cells after 1 h at 4°C; the unbound virus, Vt, was calculated as the input virus minus the number of infectious foci detected in cells infected at a given time.

To evaluate the role of FcRn and DPP4 on the infectivity of HAstV in Caco-2 cells, FcRn-knockout (FcRn-KO) and DPP4-knockout (DPP4-KO) Caco-2 cells were generated by transfection of the lentiviral vector lentiCRISPR v2-Blast to disrupt the *FCGRT* or *DPP4* genes. The *DPP4* gene expression was also knocked out in the genetic background of FcRn-KO Caco-2 cells, generating a cell line deficient in DPP4 and FcRn (double-KO) expression. The knockout phenotypes were verified by Western blot (Fig 1A), and the infectivity of HAstV serotypes 1, 2, and 8 was evaluated in these cells. It was found that the infectivity of all three strains was markedly reduced (about two logs) in the FcRn- and DPP4- KO cell lines (Fig 1B). Interestingly, the infectivity of the three serotypes in double-KO Caco-2 cells was below the detection limit of our infection assay (of about 10^2^ ffu/ml) (Fig 1B), strongly suggesting that both FcRn and DPP4 are necessary and sufficient for classical HAstV infection, at least in Caco-2 cells. In contrast, the infectivity of the divergent HAstV-VA1 was not affected in any of these cell lines, indicating that the infection of viruses in this clade depends on different cell entry factors.

### DPP4 is the receptor for classical HAstV

The cell binding of HAstV-1 was evaluated in the three knockout cell lines (Fig 1C). As previously reported [13,14], virus binding to FcRn-KO cells was not modified, indicating that the virus binds these cells via a cell receptor other than FcRn. Of interest, the binding of HAstV-1 to DPP4-KO and double-KO Caco-2 cells decreased by approximately 85% compared to wild-type (WT) or FcRn-KO cells (Fig 1C). The residual 15% binding appears to be mostly nonspecific since viral infectivity showed a reduction of approximately 2.5 logs in DPP4-KO cells and at least 5 logs in the double-KO cell line. These results suggest that despite binding to some extent to the DPP4-KO cell surface, this nonspecific interaction does not lead to a significant productive viral infection (Fig 1B). Thus, in Caco-2 cells, DPP4 seems to be the receptor for classical HAstV. At the same time, FcRn could function as a coreceptor for virus internalization and/or genome uncoating (see below).

In the reports by Haga et al. [13] and Ingle et al. [14], as well as in the assay shown in Fig 1C, the attachment of HAstV-1 to the cell surface was measured by RT-qPCR, which detects the bulk of physical virus particles. However, this assay does not distinguish between productive and nonproductive infectious pathways masked by the high ratio of physical to infectious viral particles present in most animal virus preparations [25,26]. For HAstV-1, only one in about 5,900 HAstV physical particles is infectious (see Materials and Methods). Thus, we designed an infectivity-based binding assay to determine the attachment kinetics of infectious virus, not physical particles, in WT, FcRn-KO, and DPP4-KO cells. In this assay, trypsin-activated virus was added to cells at an MOI of 0.02 and incubated in an ice-cold water bath to prevent virus entry. At different time points, the unbound virus was removed, the cells were washed twice and then shifted to 37°C to allow virus entry; 18 h later the cells were fixed and immunostained with a polyclonal antibody to the HAstV-1 capsid core domain (α-core1), and the virus-infected cells were detected by an immunocytochemistry assay as described in Material and Methods. The data were processed as previously described [11] and plotted against time. Under the conditions of this assay, the half-time of HAstV-1 binding was essentially the same in WT (8.4 min) and FcRn-KO (8.3 min) cells (Fig 1D), suggesting that both physical and infectious virus particles are not dependent on FcRn for cell binding. Unexpectedly, the low amount of infectious HAstV-1 particles detected in DPP4-KO cells (Fig 1B) interacts with these cells with a similar kinetics (9.4 min) to that observed in WT and FcRn-KO cells (Fig 1D).

### FcRn blocks HAstV-1 infectivity and cell binding in wild-type and FcRn-KO Caco-2 cells

**Fig 2:**
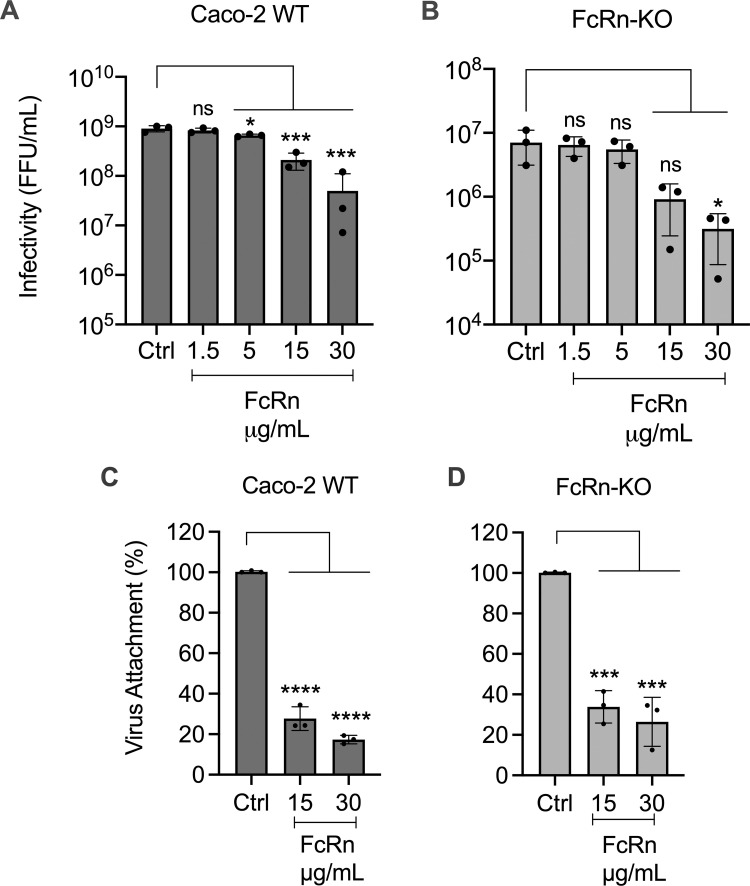
HAstV infectivity and cell binding are blocked by preincubation with soluble FcRn. (A, B), Purified, trypsin-activated HAstV-1 (160 ng/well) was incubated with the indicated concentrations of the recombinant FcRn/B2M complex for 1 h at 37°C. The infectious virus titer was then determined in WT and FcRn-KO cell monolayers grown in 96-well plates as indicated in Materials and Methods. Data are expressed as focus-forming units (ffu)/ml and represent the mean ± SD. (C, D), The indicated concentrations of recombinant FcRn ectodomain protein were incubated with purified HAstV-1 for 1 h at 37°C. The virus-protein mixtures were added to WT or FcRn-KO Caco-2 cells for 1 h at 4°C to allow the virus to attach to the cell surface. After removing the unbound virus and washing the cells, the bound virus was determined by RT-qPCR, as described in the Materials and Methods section. Experiments were performed on ice to prevent virus endocytosis. Data are expressed as percentages of the virus attached in the absence of FcRn (Ctrl) and represent the mean ± SD. The infectivity and binding assays were performed in three (A, B, C) or two (D) independent experiments, each with technical duplicates. Significance was determined by using a t-test. *, P ≤ 0.05; **, P ≤ 0.01; ***, P ≤ 0.001; ****, P ≤ 0.0001.

To evaluate if the infectivity of HAstV-1 is affected when incubated with recombinant FcRn ectodomain protein, the virus (MOI = 0.02) was pre-incubated with different concentrations of FcRn, and the virus-FcRn mix was added to either WT or FcRn-KO Caco-2 cells for 1 h at 37^o^C. After this time, cells were washed and incubated at 37^o^C for 18 h. The cells were then fixed and immunostained, and the infectious focus-forming units (ffu) were determined as described in Materials and Methods. As expected, incubation of HAstV-1 with FcRn protein inhibited infectivity in a dose-dependent manner in WT Caco-2 cells by more than one log at 30 µg/ml (Fig 2A), as described [13,14]. However, the FcRn protein unexpectedly also decreased virus infectivity in the FcRn-KO cells to a similar extent (Fig 2B).

The effect of FcRn protein on the binding of HAstV-1 was also evaluated; the virus was pre-incubated with different concentrations of FcRn for 1 h at RT, and added to either WT or FcRn- KO cell monolayers in 48-well plates and incubated with the virus-FcRn complex for 1 h on ice. The bound virus was determined by RT-qPCR as described above. Pre-incubation of the virus with FcRn reduced the attachment of the viral particles by 80%, not only in WT but also in FcRn-KO Caco-2 cells (Fig 2C and 2D). Overall, these results indicate that the interaction of FcRn with the virus particle sterically blocks its interaction with DPP4, suggesting that the binding sites for FcRn and DPP4 on the virus particle are spatially close.

### Homotypic Nt-MAbs block binding of FcRn to HAstV by ELISA

**Fig 3:**
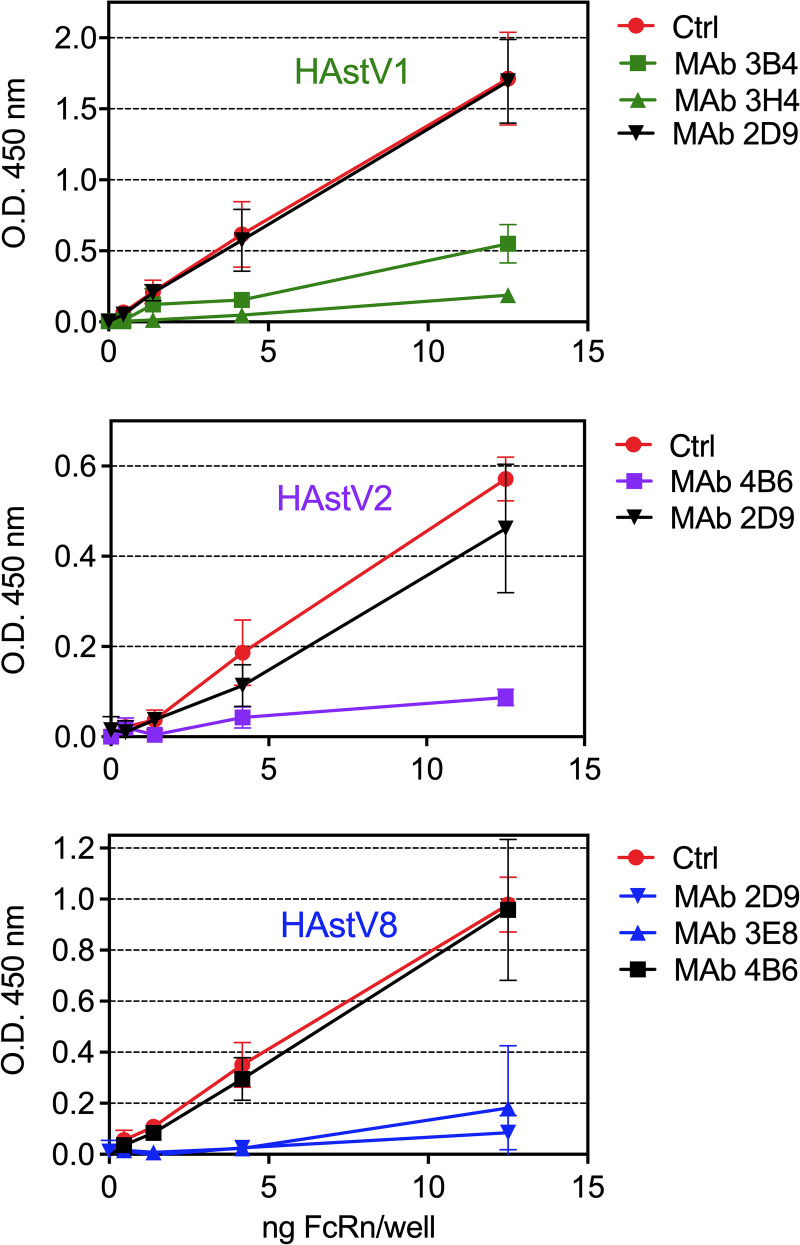
Nt-MAbs inhibit the interaction of FcRn with HAstV. Purified virus (2 μg/ml) was adsorbed for 8 h at 4°C to 96-well ELISA plates. The ascitic fluid of either Nt-MAb 2D9, 3E8, 3H4, 3B4, or 3B6 [27], diluted 1:100 in BSA/PBS, or only BSA/PBS (control), was added to the wells for 1 h at 37°C. Then, a mix of MAb (1:100) and biotinylated recombinant human FcRn heterodimer protein was added at the indicated concentrations and incubated for 1 h at 37°C. The plates were subsequently incubated for 1 h at room temperature (RT) with peroxidase-conjugated streptavidin diluted 1:4000 in PBS. After washing the wells, the reaction was developed by adding the TMB peroxidase substrate and revealed according to the manufacturer’s instructions. The assay was performed in two independent experiments for HAstV-2 and HAstV-8, or in three independent experiments for HAstV-1, and carried out in technical duplicates. The data are expressed as the optical density (O.D. at 450 nm) of the recombinant FcRn attached to the corresponding purified virus, representing the mean ± SD.

To further characterize the direct interaction of recombinant FcRn with HAstV and the effect of homotypic and heterotypic neutralizing monoclonal antibodies (Nt-MAbs) [27] on this interaction, a solid-phase assay was developed. Purified HAstV particles of serotypes 1, 2, or 8 were bound to 96-well ELISA microtiter plates. The plates were then incubated with different concentrations of biotinylated recombinant FcRn, and its binding was detected with peroxidase-labeled streptavidin. We found that the recombinant FcRn bound in a dose-dependent manner to all three viruses (Fig 3, Ctrl). Then, to test if the Nt-MAbs were able to prevent the virus-FcRn interaction, the virus adsorbed to the plates was initially incubated with serotype-specific Nt-MAbs (3B4 and 3H4 for HAstV-1; 4B6 for HAstV-2; 2D9 and 3E8 for HAstV-8). After washing the unbound antibody, biotinylated FcRn was added at the indicated concentrations, and its binding was monitored with peroxidase-labeled streptavidin. A heterotypic MAb was used as a negative control in each case (MAb 2D9 for HAstV-1 and HAstV-2; MAb 4B6 for HAstV-8). Interestingly, the virus-FcRn interaction was blocked by pre-incubation of the virus with all homotypic Nt-MAbs tested. In contrast, heterotypic Nt-MAbs, which do not cross-neutralize, did not prevent the virus-FcRn interaction (Fig 3), suggesting that Nt-MAbs inhibit the interaction of the virus with FcRn in the host cell. In contrast, heterotypic Nt-MAbs, which do not cross-neutralize, did not prevent the interaction (Fig 3).

### Nt-MAbs block the infectivity and binding of HAstV-1 to FcRn-KO and DPP4-KO Caco-2 cells

**Fig 4:**
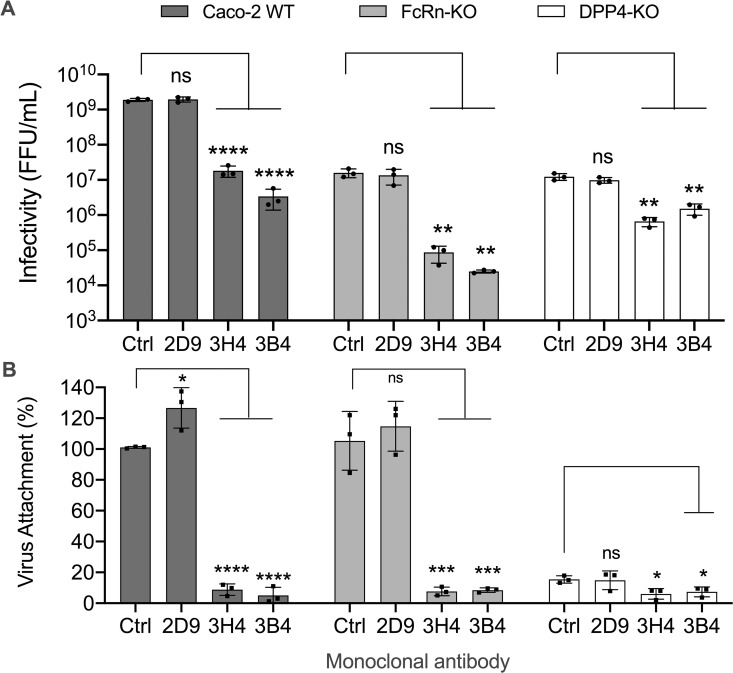
HAstV infectivity and cell binding are blocked by preincubation with neutralizing monoclonal antibodies. (A). Ascitic fluid of either Nt-MAb 3B4 or 3H4, to HAstV-1, or 2D9 to HAstV-8 (used as a negative control), diluted 1:100, was incubated with purified, trypsin-activated HAstV-1 (at an MOI of 0.02) for 1 h at 37°C. The virus-antibody mixtures were adsorbed for 1 h at 37°C to WT, FcRn-KO, or DPP4-KO Caco-2 cell monolayers grown in 96-well plates. After removing the unbound virus and washing the cells, the cultures were kept for 18 h at 37°C, and the cells were then fixed and immunostained for infectious foci as described in Materials and Methods. Data are expressed as focus-forming units (ffu)/ml and represent the mean ± SD. (B) Ascitic fluid of either MAb 3B4, 3H4 to HAstV-1, or 2D9, diluted 1:100, was incubated with 160 ng of purified, trypsin-activated HAstV-1 for 1 h at 37°C. The virus-antibody mixtures were added to the indicated cell lines for 1 h at 4°C to allow the virus to attach to the cell surface. After removing the unbound virus and washing the cells, the bound virus was quantified using RT-qPCR, as described in the Materials and Methods section. Experiments were performed on ice to prevent virus endocytosis. Data are expressed as percentages of the virus attached in the absence of MAbs (Ctrl) and represent the mean ± SD. The infectivity and binding assays were performed in three independent experiments carried out in technical duplicates. Significance was determined by using a t-test. *, P ≤ 0.05; **, P ≤ 0.01; ***, P ≤ 0.001; ****, P ≤ 0.0001).

We have found that the Nt-MAbs: i) neutralize the infectivity and the attachment of classical HAstVs in Caco-2 cells [28] and ii) block the interaction of FcRn with the virus particle in vitro (see above). We also showed that the sites on the virus particle that interact with FcRn and DPP4 seem to be structurally close (see above). Considering these findings, we evaluated whether the Nt-MAbs would interfere with the infectivity and attachment of HAstV-1 to FcRn-KO and DPP4-KO Caco-2 cells. As shown in Fig 4A, when tested at a 1:100 dilution, ascitic fluids of MAbs 3B4 and 3H4, specific for HAstV-1, reduced the infectivity of HAstV-1 in WT and FcRn Caco-2 cells by 2–3 logs. In contrast, the infectivity in DPP4-KO Caco-2 cells decreased only by 8- to 18-fold (Fig 4A), supporting the idea that HAstV-1 binds a nonspecific ligand in DPP4-KO cells, and most likely it does so through a site on the spike close, but different from the sites recognized by Nt-MAbs 3B4 and 3H4. As expected, the HAstV-8-specific MAb 2D9 did not affect virus infection in any of the three cell lines.

On the other hand, to evaluate whether the Nt-MAbs prevent the interaction of the virus with DPP4 and FcRn in cells in culture, we tested whether they affected the binding of the virus to WT, FcRn-KO, and DPP4-KO cells. As previously reported [28], the homotypic MAbs 3B4 and 3H4, but not the heterotypic MAb 2D9, efficiently blocked HAstV-1 binding to WT Caco-2 cells (Fig 4B). Unexpectedly, the homotypic MAbs also blocked virus attachment to Caco-2 FcRn-KO cells to about 10% that of the control (2D9 MAb), indicating that the homotypic MAbs can neutralize virus infectivity in these cells by preventing the interaction of the virus with DPP4. On the other hand, MAbs 3B4 and 3H4 blocked virus binding in Caco-2 DPP4-KO cells to about 50% of the attachment observed in the presence of the control 2D9 MAb or the absence of antibody (Fig 4B). This finding is consistent with the observation that virus binding to DPP4-KO cells appears to be nonspecific (Fig 1C) and suggests that this nonspecific interaction occurs at a site on the virus spike that partially overlaps the recognition site of the homotypic MAbs, although to a less degree compared to the site on the spike that interacts with DPP4. Altogether, these results reinforce the idea that the virus interaction sites with FcRn and DPP4 are likely to be very close spatially, and that Nt-MAbs sterically interfere with both interactions.

### Isolation of HAstV mutant viruses with lower affinity to FcRn

**Fig 5:**
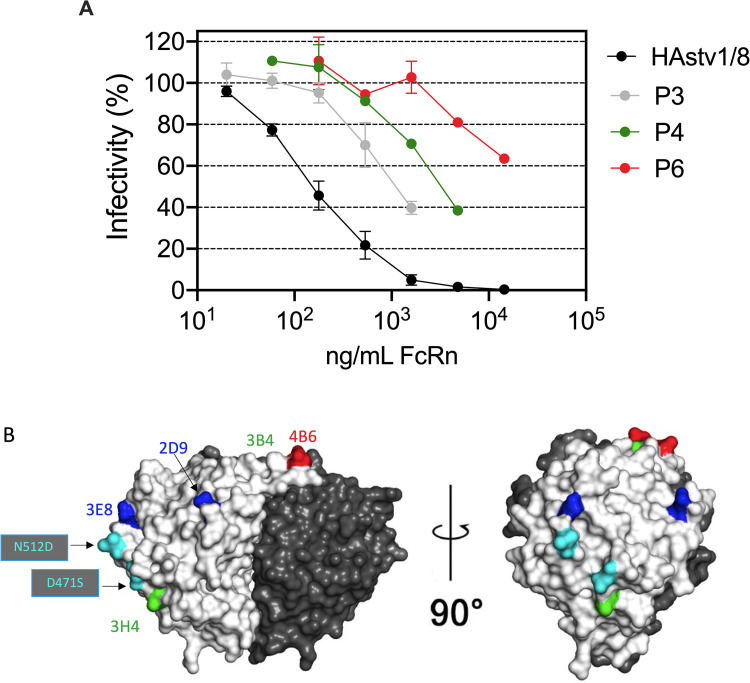
Mutations in the HAstV1/HAstV-8 chimeric virus were found in the FcRn-resistant variants. (A) Trypsinized HAstV1/HAstV-8 chimeric virus was incubated with recombinant FcRn (8 µg/ml) for 1h at 37^o^C. The virus-FcRn mix was used to infect Caco-2 cells for 24 h at 37^o^C. Cell lysates were then prepared by three freeze-thawing cycles, and the produced virus was incubated again with FcRn. The selection procedure was repeated until the detection of FcRn-escape variants whose infectivity was less susceptible to inhibition by FcRn. The infectivity assay was performed in biological triplicate and in technical duplicates for the chimeric HAstV-1/8 virus; in a single replica and technical triplicate for P3; and in a single replica, in technical duplicate for P4 and P6. Data are expressed as percentages of the HAstV1/HAstV-8 chimeric virus infectivity obtained in the absence of FcRn and represent the mean ± SD. (B). Localization of the point mutations that confer resistance to FcRn infectivity inhibition. The HAstV1/HAstV-8 chimeric virus capsid protein sequence from passage six in the presence of FcRn was determined and compared to the WT HAstV-8 virus sequence. The crystal structure of the HAstV-2 spike (PDB code 5KOU) was used as a scaffold to show the HAstV neutralization escape mutations by all Nt-MAb and FcRn. The spike is shown in two orientations. One half of the dimer is colored dark gray, and the other half is light gray. The mutations that allow HAstV-8 to escape recombinant FcRn neutralization are colored in cyan. The mutations that allow classical HAstV to escape neutralization by Nt-MAbs are shown as a reference for the location of the FcRn escape mutations. They are in green for MAbs 3B4 and 3H4 to HAstV-1; in blue for MAbs 3E8 and 2D9 to HAstV-8; and in red for MAb 4B6 to HAstV-2. The mutations are shown in only one protomer of the dimer.

To define the site on the virus capsid spike recognized by FcRn, we explored the possibility of isolating virus variants resistant to the inhibition caused by the recombinant FcRn protein, similar to the isolation of mutant viruses that escape neutralization by Nt-MAbs [27,29]. For this, we incubated a lysate of cells infected with a chimeric HAstV-1/HAstV-8 virus (see Materials and Methods and ref [30]) with recombinant FcRn (8 µg/ml) for 1 h at 37^o^C. The virus-FcRn mix was used to infect Caco-2 cells for 24 h at 37^o^C. Cell lysates were obtained by three freeze-thawing cycles, and the resultant lysate was again incubated with FcRn protein and used to infect a new cell monolayer. The selection procedure was repeated until the detection of escape variants whose infectivity was less susceptible to inhibition by FcRn. The FcRn-resistant phenotype of the virus population after various passages was evaluated using a neutralization assay with different concentrations of FcRn (Fig 5A). We detected virus variants whose infectivity was less inhibited by FcRn after three passages in the presence of FcRn. This resistance increased in the sixth passage (Fig 5A); while the infectivity of the WT virus decreased 50% at about 100 ng/ml of FcRn, the infectivity of the FcRn-resistant virus population in the sixth passage completely escaped neutralization by 1 µg/ml of FcRn, and a 50% inhibition was achieved at 10 µg/ml of FcRn. The sequence of the region encoding the capsid protein of this FcRn-resistant virus population was determined and compared to the sequence of the wild-type (WT) virus. Two amino acid changes were identified in the FcRn-resistant virus. A change of Asp to Ser at position 471, and a second change of Asn to Asp at amino acid residue 512. These two mutations lie on the surface of the virus spike, in the middle and most distal region of the protomer-protomer interface (Fig 5B). Of interest, these two changes map close to previously identified mutations that allow HAstV-1 (K504E) and HAstV-8 (Y464H) to escape neutralization by Nt-MAbs 3H4 and 3E8, respectively (Fig 5B and [27]), suggesting that they might indeed be part of the binding site of FcRn in the two protomers of the virus spike dimer.

**Fig 6:**
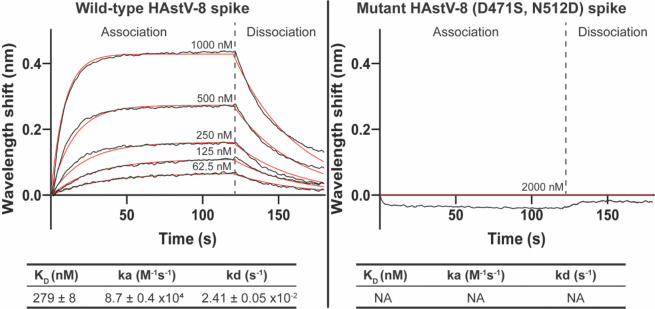
HAstV-8 (D471S, N512D) spike has decreased or no binding to FcRn compared to wild-type HAstV-8 spike. Representative biolayer interferometry traces of HIS1K biosensors loaded with WT HAstV-8 spike (left) or mutant HAstV-8 (D471S, N512D) spike (right) and dipped into a 2-fold dilution series of FcRn ranging from 1000 to 62.5 nM. Due to a lack of binding, only one concentration of FcRn (2000 nM) is shown for the mutant HAstV-8 (D471S/N512D) spike. Binding signals are shown as black lines, and curve fits are shown as red lines. Kinetics (K_D_, ka, and kd) are reported as the average of three replicates ± SD.

To evaluate the effect of the point mutations found in the FcRn-resistant HAstV-1/HAstV-8 chimeric virus, we expressed and purified a recombinant WT HAstV-8 spike or a mutant spike bearing the two mutations identified, D471S and N512D. The affinity of the WT and mutant HAstV-8 spikes was determined by biolayer interferometry (BLI). Biosensors coated with either spike protein were dipped into wells containing 1:2 serially diluted recombinant FcRn in assay buffer to determine the association rate. Biosensors were then dipped in assay buffer to determine the dissociation rate. We found that the WT HAstV-8 spike has a dissociation constant (K_D_) of about 280 nM (Fig 6). In contrast, the mutant HAstV-8 (D471S, N512D) spike did not bind to FcRn at concentrations up to 2,000 nM FcRn, indicating that its binding capacity to FcRn was eliminated or significantly decreased, suggesting that the identified mutations are located in the spike region recognized by FcRn.

### The neonatal FcRn receptor facilitates HAstV internalization and uncoating of infectious virus

**Fig 7:**
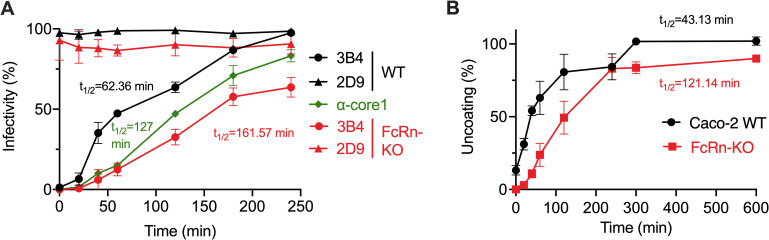
HAstV-1 internalization and uncoating are delayed in FcRn-KO Caco-2 cells. (A) HAstV-1 at an MOI of 0.02 was added to WT or FcRn-KO Caco-2 cells for 1 h at 4ºC. The cells were switched to 37ºC and incubated for the indicated times to allow virus internalization. To remove the virus adsorbed to the cell surface that had not been internalized at different time points, the cells were incubated with Nt-MAb 3B4 ascitic fluid diluted 1:50 for 1 h at 4 ºC, and the infection was left to proceed for 18 h at 37 ºC. After this period, the cells were fixed and immunostained for infectious foci as described in the Materials and Methods section. This assay was performed in three independent experiments and in technical duplicates. The data are expressed as percentages of the HAstV-1 infectivity obtained in the absence of MAb 3B4 at time 0, which was taken as 100%, and represent the mean ± SD. (B) HAstV-1 genome uncoating is delayed in FcRn-KO cells. A neutral red-labeled HAstV-1 virus stock protected from white light was prepared as described in Materials and Methods. To evaluate the kinetics of viral genomic RNA release, two-fold dilutions of the neutral red-HAstV-1 lysate were adsorbed to cells for 1 h at 4°C in the dark, and the cultures were then shifted to 37°C and exposed to white light at the indicated times post-adsorption; the cells were kept for 18 h at 37°C, at which time the cells were fixed and immunostained for infectious foci as described in Materials and Methods. The infectivity assay was performed in three independent experiments and in technical duplicates. The data are expressed as percentages of HAstV-1 infectivity observed in the neutral red-labeled virus exposed to light compared to the same virus kept in the dark and represent the mean ± SD.

Given that FcRn is not required for the binding of the virus to the cell surface, we explored its role in promoting cell internalization and uncoating of the viral genome. We evaluated the internalization kinetics of infectious HAstV-1 in WT and FcRn-KO Caco-2 cells using the infectivity assay previously reported [11]. Briefly, HAstV-1 (MOI = 0.02) was adsorbed to confluent Caco-2 cell monolayers for 1 h at 4^o^C to prevent virus endocytosis. After this time, the unbound virus was removed, and the cell monolayers were switched to 37^o^C for the times indicated in Fig 7A to allow the virus to penetrate the cell. To neutralize the virus adsorbed to the cell surface that had not been internalized during the incubation period at 37^o^C, the cells were incubated with a 1:50 dilution of ascitic fluid of Nt-Ab 3B4, homotypic for HAstV-1 [27], for 1 h at 37^o^C. After washing, the cells were then incubated for 18 h at 37^o^C to allow the infection of the already internalized virus to proceed. After this time, the number of infectious foci was determined as described in Materials and Methods. At the dilution used, MAb 3B4 neutralized all infectious virus bound to the cell surface that had not yet been endocytosed, as observed when the antibody was added just after the end of the adsorption period (time 0 in Fig 7A). As a negative control, ascitic fluid of the heterotypic Nt-MAb 2D9 directed to HAstV-8 was used. Of interest, the half-time of internalization of infectious HAstV-1 in FcRn-KO Caco-2 cells (t_1/2_ = 162 min) was 2.6 times slower than in Caco-2 WT cells (t_1/2_ = 62 min) (Fig 7A). In addition, 4 h after shifting the cell culture to 37^o^C, 100% of the infectious virus added to WT Caco-2 cells had reached a cell compartment (presumably endocytic vesicles) that allowed the virus to escape neutralization at the cell surface, since all attached viruses escaped antibody neutralization. On the other hand, in FcRn-KO cells, only about 60% of the infectious virus had been internalized after 4 h at 37^o^C (Fig 7A). These results suggest that the neonatal Fc receptor facilitates the internalization and the efficiency of infection of HAstV-1 since the infectivity of the virus in WT cells was about two logs lower than in FcRn-KO cells (Fig 1B).

We next examined the potential role of FcRn in the final step of the virus entry process, specifically the uncoating of the viral genome that occurs when the virus releases the viral genome into the cytoplasm from the endocytic vesicle. To determine the time at which HAstV-1 uncoats in WT or FcRn-KO Caco2 cells, a neutral red-based RNA release assay was carried out, as described [11]. This assay has been used to evaluate the RNA release of different single-stranded positive-RNA viruses, such as poliovirus [31,32] and calicivirus [33]. This method is based on the fact that the neutral red is incorporated into the viral particles during virus replication. The proximity of the dye to the viral RNA makes the virus infectivity sensitive to white light exposure; however, once the genomic RNA is released into the cytoplasm, the dye diffuses out, and the infection can proceed in white light [11,31,32]. For these assays, we prepared a virus lysate by infecting Caco-2 cells with HAstV-1 in the presence of neutral red, in the dark. The dye-loaded virus (MOI = 0.02) was adsorbed to WT or FcRn-KO Caco-2 cells in the dark for 1 h at 4^o^C. After this time, the cells were shifted to 37^o^C, and kept in the dark. At different times after the adsorption period (Fig 7B), cells were exposed to light, and the infection was left to proceed for 18 h at 37^o^C under regular white light conditions. The infectivity was then determined as described in the Materials and Methods section. Of interest, and similar to the internalization time, the kinetics of HAstV-1 uncoating in FcRn-KO cells was delayed as compared to WT Caco-2 cells (Fig 7B). The half-time for RNA release in FcRn-KO cells was estimated to be about 121 min, 2.8-fold slower than in WT cells (t_1/2_ = 43 min). Similar to the internalization assay, the RNA uncoating process was incomplete in FcRn-KO cells, reaching only 90% of the initially added infectious virus at 10 h post-infection (hpi). These results suggest that FcRn could also be relevant for HAstV-1 RNA release, in addition to its role in virus internalization.

### Virus internalization in FcRn-KO cells leads to a low productive infection

**Fig 8:**
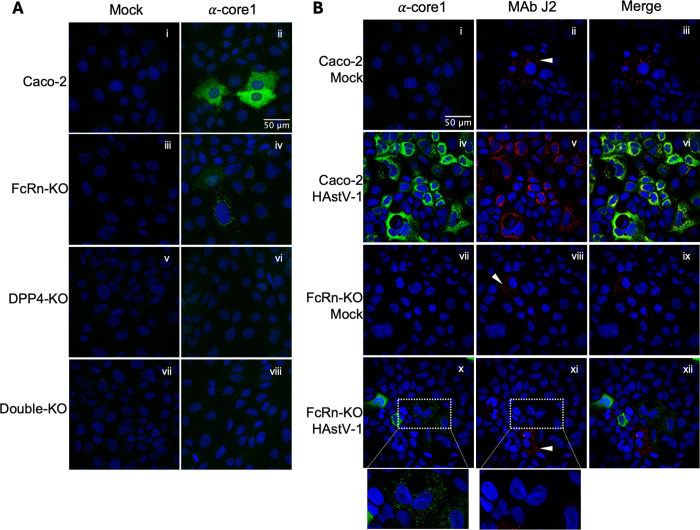
Immunofluorescence analysis of viral proteins in KO cells. A) Caco-2 cells were infected with HAstV-1 at an MOI of 3 or mock-infected. At 8 hpi, the cells were fixed with formaldehyde, permeabilized with Triton X-100, and incubated overnight at 4^o^C with a polyclonal antibody to the HAstV-1 capsid core protein [27]. Cells were subsequently incubated with Alexa 488-labeled anti-rabbit IgG antibodies for 1 h at RT and observed by confocal IF microscopy at 60X magnification, as described in Materials and Methods. Nuclei were stained with DAPI. (B) Caco-2 cells were infected with HAstV-1 as described in (A), and after fixation and permeabilization, they were incubated overnight at 4^o^C with a mixture of the HAstV-1 capsid core antibody and MAb J2 to dsRNA. Cells were subsequently incubated with a mixture of Alexa 488-labeled anti-rabbit IgG (green) and Alexa 568-labeled anti-mouse IgG antibodies (red) for 1 h at RT and observed by confocal microscopy as described in Materials and Methods. In all panels, the nuclei stained with DAPI are shown. The white arrowheads (panels ii, viii, and xi) show uninfected cells with punctuated MAb J2 signal.

To further explore HAstV-1 internalization, we analyzed the presence of viral antigen at 8 hpi in WT and FcRn-KO cells by immunofluorescence (IF) confocal microscopy. For comparison, we included DPP4-KO and double-KO Caco-2 cells in the analysis. The rationale was that at 8 hpi we could distinguish cells with internalized, but not uncoated virus, from cells in which a productive virus infection was taking place, i.e., showing viral protein synthesis in the cytosol. In these assays, WT, FcRn- DPP4-, and the double-KO Caco-2 cells were infected with HAstV-1 at an MOI of 3, for 8 h at 37^o^C. Cells were then fixed with paraformaldehyde, permeabilized with Triton-X-100, and stained with polyclonal antibodies to the HAstV-1 core capsid protein as described in Materials and Methods. We observed two different antibody staining patterns in the infected cells that were not present in mock-infected cells. The first showed an intense cytoplasmic signal that most probably reflects cells with *de novo* viral protein synthesis in the cytoplasm (Fig 8A, panel ii). The second pattern consisted of a punctuated IF signal that probably represents internalized virions whose genomes have not been released into the cytoplasm and remain in endocytic vesicles (Fig 8A, panel iv). The pattern of infected cells was observed in WT and FcRn-KO Caco-2 cells, although in quite different proportions. Of 1,429 and 1,160 WT and FcRn-KO cells analyzed (average of three different experiments), respectively, the infected pattern was observed in 18.5% of the WT and 0.35% of the FcRn-KO cells (52:1 ratio). In contrast, cells with a punctuated pattern were not detected in WT cells, whereas they represented 4.71% of the FcRn-KO cells. These two staining patterns were not observed in either DPP4-KO or the double-KO cells (Fig 8A), most likely because the virus binds poorly to these two cell lines (Fig 1C).

To evaluate if the punctuated IF signal observed in Fig 8A, panel iv, represents virus replication centers in FcRn-KO Caco-2 cells, we stained the cells with the monoclonal antibody J2, which has been shown to specifically detect the astrovirus dsRNA genome replication intermediates [34]. We detected astrovirus replication centers in WT Caco-2 cells at 8 hpi infected with HAstV-1, identified by the colocalization of the J2 and anti-HAstV core antibody signal (Fig 8B, panel vi). On the other hand, this colocalization was not observed in the punctuated signals observed in FcRn-KO cells (Fig 8B, panel xii), supporting the idea that the puncta observed with anti-HAstV antibodies might reflect incoming virus particles trapped in endocytic vesicles. Of interest, in mock-infected WT and FcRn-KO cells, some cells showed a punctuated pattern when incubated with antibody J2 (Fig 8B, panels ii and viii, white arrowheads). A similar pattern was also occasionally observed in uninfected WT and FcRn-KO cells that had been previously infected (Fig 8B, panel xi). The reason for this signal in uninfected cells is unknown, but it does not correlate with HAstV infection. Altogether, these results strongly suggest that the virus can be internalized in both WT and FcRn-KO cells. However, the viruses that penetrate the cell by the FcRn-mediated pathway frequently, if not mostly, lead to a productive infection. This also implies that the FcRn-independent internalization observed in FcRn-KO cells is a quasi-dead end for potentially infectious virions, with only some viruses able to infect the cell.

## Discussion

Recently, two publications reported the identification of the human neonatal Fc receptor as a functional receptor for HAstV in Caco-2 cells [13,14], and one of them reported dipeptidyl peptidase 4 as an entry factor [14]. FcRn-KO of enteroids [13] or incubation of enteroids with pharmacological inhibitors of DPP4 or FcRn activities [14] decreased HAstV replication, indicating the relevance of these molecules for HAstV infection of non-cancerous enteric cells. Both groups demonstrated that FcRn expression promotes HAstV infection in low- or non-permissive cells and reported direct binding of infectious HAstV particles and recombinant HAstV capsid spike to FcRn. In contrast, while DPP4 expression promotes HAstV infection in non-permissive cells, neither the recombinant HAstV capsid spike nor the capsid core was found to bind DPP4; however, the complete virus particle was not tested in these assays. Thus, the molecular basis for HAstV interaction with FcRn and the mechanistic basis for the roles of FcRn and DPP4 in HAstV infection were not completely defined. Regarding the role of DPP4, our findings demonstrated that HAstV-1 binding is significantly reduced in DPP4-KO and DPP4-KO/FcRn-KO Caco-2 cells (Fig 1C), strongly suggesting that DPP4 functions as the receptor for classical HAstV in Caco-2 cells. However, whether DPP4 interacts directly with the virus or indirectly through one of its multiple cellular binding partners, such as adenosine deaminase [35,36], remains unclear. Additionally, the interaction might occur via the glycan moiety of the DPP4 dimer, as recently reported for porcine hemagglutinating encephalomyelitis virus [23].

Notably, in the publications by Ingle et al. [14] and Haga et al. [13], as well as in this work (Fig 1), HAstV were found to bind to FcRn-KO Caco-2 cells to a degree similar to that observed in WT cells. In all cases, the binding of the virus was detected by RT-qPCR, an assay that detects physical virus particles attached to the cell surface. However, assays that detect physical particles generally require a very high multiplicity of infection (MOI), increasing the probability of measuring nonproductive pathways [25], due to the large proportion of noninfectious virions present in any virus preparation, and thus, the interpretation of the results of any biochemical experiments performed on a virion population is subject to the assumption that the biochemical properties of the infectious subpopulation are accurately represented. In the case of poliovirus, the particle/PFU ratio can be in the thousands (Kirkegaard, 1990 #5). For rotavirus, the reported ratio is 100:1–30,000:1 [37], and in general, for most animal viruses, it is thought to be in the range of several million to 10 [38]. In this work, we determined a physical-to-infectious virus particle ratio of approximately 5,900 for HAstV-1. In this context, we wondered if the binding of infectious virus particles, as opposed to physical virus particles, could have been obscured in FcRn-KO cells when RT-qPCR measured the bulk of viruses. To evaluate this possibility, we determined the kinetics of attachment of infectious virus in FcRn-KO and WT cells. As shown in Fig 1D, the half-time of attachment of HAstV-1 to these two cell lines was not significantly different, between 8 and 9 min, and similar to the previously reported half-time of attachment for HAstV-8 of 9.2 min in Caco-2 cells [11]. These results suggest that the infectious virus does not utilize FcRn for cell attachment, but rather most likely uses DPP4, as is the case for the bulk of virus particles. We also evaluated the kinetics of the residual binding of the virus to double-KO Caco-2 cells. Surprisingly, infectious HAstV-1 particles attached to these cells with a half-time (9.3 min) similar to that observed in WT and FcRn-KO cells (Fig 1D), suggesting a rapid interaction of the infectious virus with an unknown cell ligand that very inefficiently promotes virus infection (Fig 1B).

On the other hand, we found that preincubation of recombinant soluble FcRn with HAstV-1 prevents the attachment and the infectivity to WT Caco-2 cells, but notably, also to FcRn-KO cells (Fig 2B and 2D), suggesting that the FcRn binding site on the virus capsid spike is spatially located close to the site used by the virus to attach to DPP4 on the cell surface. Furthermore, Nt-MAbs block the interaction of FcRn with the virus (Fig 3) as well as the infectivity and binding of HAstV-1 to both WT and FcRn-KO Caco-2 cells (Fig 4). Altogether, these results reinforce the idea that the sites on the virus spike that interact with FcRn and DPP4 are located very close to each other on the spike surface. Considering the observation that homotypic Nt-Abs can prevent both interactions, this seems to represent a functional domain in the virus capsid spike. To locate this relevant region, we generated HAstV-8 variants with a decreased ability to interact with FcRn (Fig 5). Characterization of the spike amino acid sequence of the variant population identified two amino acid changes, D471S and N512D, which map close to each other on the spike surface. Of interest, the D471S mutation is spatially very close to the Y464H mutation (Fig 5B) in the HAstV-8 spike, allowing the virus to escape neutralization by MAb 3E8 [27]. In fact, Tyr464 was found to be buried by the footprint of antibody 3E8 when the X-ray crystal structure of the HAstV-8 spike-antibody 3E8 complex was determined [28]. Similarly, mutation N512D lies close to the escape mutation K504E, which prevents HAstV-1 from being neutralized by MAb 3H4 [27], and amino acid residue Lys504 also resides on the antibody 3H4 footprint in the X-ray crystal structure of the HAstV-1 spike-antibody 3H4 complex [28]. These findings suggest that the sites recognized by FcRn and by antibodies 3E8 and 3H4 overlap. The relevance of amino acid residues Asp471 and Asn512 for the interaction of FcRn and the HAstV-8 spike was confirmed by the significant loss of interaction of FcRn with a recombinant mutant HAstV-8 spike bearing the two amino acid changes (Fig 6). Lastly, and most notably, amino acid residues equivalent to Asp471 and Asn512 in the HAstV-1 spike were recently identified as critical for the FcRn-spike interaction by X-ray structure analysis of the HAstV-1 spike-FcRn complex (Rebecca DuBois, personal communication).

HAstV-8 has been shown to enter cells by clathrin-mediated endocytosis [11]. Since antibody internalization by FcRn can also occur through this pathway [39–41], and it does not mediate the attachment of the virus to cells (Fig 1), we hypothesize that the primary role of FcRn during HAstV infection could be to facilitate the endocytic internalization of the virions. Our findings support this role for FcRn, since the internalization time of HAstV-1 in FcRn-KO cells, using an infectious virus assay, was found to be delayed 2.6-fold compared to WT Caco-2 cells (162 vs 62 min), and by the observation that the internalization in FcRn-KO cells is not complete even after 4 h of incubation at 37^o^C (Fig 7A). Also, interestingly, we found that the half-time for HAstV-1 genome uncoating was 43 min in WT Caco-2 cells, while in FcRn-KO cells there was a 3-fold lag (Fig 7B). These findings suggest that FcRn could also be involved in the final step of the virus entry process.

Of note, the half-time of HAstV-1 internalization determined in this work in WT Caco-2 cells was longer than that of RNA release (62 vs. 43 min). These paradoxical results could be explained by the different nature of the two assays. Despite both having a readout of infectivity, in the internalization assay, the surface-exposed virus was neutralized with a high concentration of Nt-MAb for 1h at 37^o^C, which somehow could delay the cell entry process and the detection of infected cells in the infectivity assay. This interpretation is in line with the fact that using a 1:50 serum dilution of a polyclonal antibody to the HAstV-1 spike instead of MAb 3B4 during the internalization kinetics resulted in an even longer half-time of internalization of 120 min (green line in Fig 7A). To correctly establish the internalization time of the virus would require inactivating the surface-exposed virus by chemical or biochemical methods different from the use of neutralizing antibodies. Nonetheless, the internalization and uncoating times of the virus are delayed in FcRn-KO cells, indicating that the absence of FcRn influences these two steps. As a reference, the same neutral red assay was used to determine the murine norovirus half-time of RNA release of 33 min in murine macrophages [33], and with poliovirus in HeLa cells, which showed a half-time of RNA release of 18 min [31].

We hypothesize that the binding of mature, trypsinized HAstV to FcRn in a late, low-pH endosome environment triggers the previously described membrane disrupting activity associated with the capsid core [42], enabling RNA genome release into the cytoplasm. FcRn has been previously reported as a binding receptor for different echovirus serotypes [18], and it has also been found to function as an uncoating receptor for a large group of echoviruses after binding to other cellular molecules, such as CD55 [19]. More recently, FcRn was found to have a dual function during cell entry for echovirus 18, working both in the attachment and uncoating steps [17]. In addition to facilitating virus infection in the Enterovirus B group, FcRn has also been reported to serve as a receptor for the enveloped arteriviruses [20,21]; however, its precise role during cell entry of this group of viruses has not been determined.

**Fig 9:**
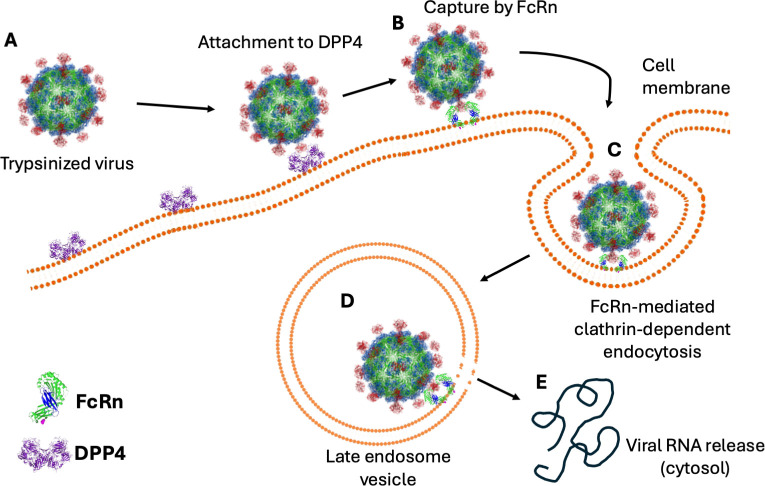
Working model for classical HAstV entry into Caco-2 cells. (A) The trypsinized virus attaches to the cell surface through the viral spike via the dipeptidyl peptidase 4 (DPP4). This interaction can occur directly or indirectly through one of this enzyme’s several binding partners on the cell surface [35,36]. (B) The virus is transferred to the neonatal Fc receptor, FcRn. The sites on the spike that interact with DPP4 are spatially close to the site recognized on the spike by FcRn. (C) The virus-FcRn interaction promotes the cell internalization of the virus particle by clathrin-mediated endocytosis [11]. (D) The virus reaches late endosomes, and the low pH, probably together with other cellular cues, promotes a structural change in the core capsid protein that promotes a lipolytic activity [42]. (E) This change may form a portal-like assembly that releases the genome into the cytoplasm.

In summary, our findings reveal that classical HAstVs utilize a two-step entry process in which DPP4 acts as a receptor, while FcRn functions as a coreceptor, facilitating the virus clathrin-dependent endocytic process and likely contributing to the viral genomic RNA release within endocytic vesicles (Fig 9).

Our laboratory’s ongoing investigations aim to further characterize the roles of these and other cellular factors in Caco-2 cells and in enteroids. These studies are expected to provide a more detailed understanding of this significant human pathogen’s early stages of infection.

## Materials and methods

### Cells and viruses

Cells from a human colon adenocarcinoma (Caco-2), clone C2BBe1, obtained from the American Type Culture Collection (ATCC, Manassas, VA, USA; CRL-2102) were used in this work. Cells were grown in DMEM Advanced (Gibco #12491015) supplemented with 5% fetal bovine serum (ByProductos #090020) and glutamine 2 mM in a 10% CO_2_ atmosphere at 37°C. Human astrovirus serotypes 1 (HAstV-1, strain Oxford), 2 (HAstV-2, strain Oxford), and 8 (HAstV-8, strain Yuc-8) were grown in Caco-2 cells [27]. Stocks of these viruses were prepared in Caco-2 cells, as previously described [43]. Briefly, the virus was activated with 200 µg/ml of trypsin for 1 h at 37ºC, and soybean trypsin inhibitor (Sigma #T9003) at 200 µg/ml was added immediately before inoculating cells. The virus was adsorbed for 1 h at 37ºC, and after this time, the inoculum was removed, and the infection was left to proceed for 18 h at 37ºC.

### Calculation of the oxford HAstV-1 infectious to physical particle ratio

HAstV-1 Oxford strain (GenBank accession number L23513.1) was purified by CsCl isopycnic centrifugation as previously described [9]. The virus protein concentration was determined by SDS-PAGE and Coomassie blue staining, using a BSA standard curve and linear regression of the densitometric analysis of the intensity of BSA and viral proteins. The calculated concentration of the virus was 80 ng/µl. Considering 180 copies of VP34 (37,063 Da), 60 copies of VP27 (27,098 Da), and one copy of the virus RNA genome (6771 nucleotides; 2,178.56 kDa) per mature, trypsinized virion, the molecular mass of one physical particle is 1.048 x 10^7 daltons. Thus, 1 ng of purified virus equals 5.746 × 10^7 particles. In our hands, 1 ng of purified HAstV-1 yielded 9.62 × 10^3 ffu. Therefore, the physical to ffu virus particles ratio was 5,900.

### Expression and purification of HAstV-8 capsid spike in *E. coli*

The capsid spike domain sequence for WT HAstV-8 and mutant HAstV-8 (D471S, N512D) were individually cloned into a pET52b+ expression vector in-frame with a C-terminal thrombin cleavage site and a 10x histidine affinity tag (Sigma #71554). The plasmids were heat-shock transformed into T7 Express *E. coli* cells (New England Biolabs #C2566H). A culture was grown in LB media with ampicillin via shaking at 37^o^C. Upon reaching an OD_600_ of 0.6, isopropyl β-d-1-thiogalactopyranoside (IPTG) was added to a final concentration of 1 mM and the culture was shifted to 18^o^C and shaken overnight. Cells were harvested by centrifugation and resuspended in lysis buffer (20 mM Tris pH 8.0, 300 mM NaCl, 20 mM imidazole). Cells were lysed by sonication and lysates were centrifuged for 30 min at 40,000g at 4^o^C. The soluble fraction was filtered through a 0.2 μm Acrodisc filter (Pall #4652) and loaded onto a gravity column with HisPur cobalt resin (ThermoFisher #89964). The column was washed with lysis buffer, and proteins were eluted by the addition of lysis buffer containing 500 mM imidazole. The sample was dialyzed into TBS (10 mM Tris pH 8.0, 150 mM NaCl) overnight at 4^o^C. Purity was assessed by SDS-PAGE with Coomassie blue staining.

### Expression and purification of FcRn in CHO-S cells

Codon-optimized synthetic genes encoding the ectodomain of the FCGRT gene (UniProt: P55899, Met1-Ser297) or the β-2-Microglobulin (β2M) gene (UniProt: P61769, Met1-Met119) were cloned separately into a modified pCDNA3.1 vector with methylation targets in the CMV promoter mutated [44]. The FCGRT construct was cloned in-frame with a C-terminal thrombin cleavage site, TwinII-Strep tag, and AviTag. Both plasmids were maxiprepped (Machery-Nagel). CHO-S cells were resuspended to a density of 2 × 10^^8^ cells/ml and electroporated with a MaxCyte STx using OC-400 cuvettes. 120 μg of DNA was used with a 1:2 (w/w) ratio of FCGRT to β2M plasmid DNA. The cells were grown in CD-OptiCHO media (Gibco #12681029) with 1 mM sodium butyrate and fed daily with CHO feed (Gibco #A1023401) supplemented with 7 mM L-glutamine, 5.5% glucose, and 23.4 g/L yeastolate. The cells were maintained at 32^o^C, 8% CO_2_, 85% humidity, and 135 rpm for 7 days. The cells were centrifuged, and the media were supplemented with 1x protease inhibitor cocktail (Millipore #539137). The media was diluted 1:2 (v/v) with StrepTrap XT binding buffer (100 mM Tris pH 8.0, 150 mM NaCl, 1 mM EDTA) and loaded onto a Cytiva StrepTrap XT affinity column. The column was washed with binding buffer, and the protein was eluted with binding buffer containing 50 mM biotin. The sample was dialyzed into TBS (10 mM Tris pH 8.0, 150 mM NaCl) overnight at 4^o^C. Purity was assessed by SDS-PAGE with Coomassie blue staining.

### Virus infectivity assay

The infectious titer of the virus stocks was determined in monolayers of 100% confluent Caco-2 cells grown in 96-well plates (seeded at 30,000 cells/well and used 48 h later). For this, cells were infected, as described above, with serial two-fold dilutions of the virus stock. After 18 h of incubation at 37ºC, the cell monolayers were processed for an immunocytochemistry (immunoperoxidase) assay. Briefly, the cells were fixed with 2% paraformaldehyde in PBS for 20 min at RT and then washed twice with PBS. The cell membrane was permeabilized with 0.2% Triton X-100 in PBS for 15 min at RT. The viral antigen was detected with serotype-specific rabbit polyclonal antibodies against the capsid spike protein of HAstV-1 (dil. 1:1,000), HAstV-2 (dil. 1:1,000) or HAstV-8 (dil. 1:2,000), as described [27], followed by incubation with peroxidase-conjugated Protein A (Calbiochem #539253) diluted 1:3000. Finally, the cells were washed twice with PBS and stained with 50 µl of a solution containing 4 mg/ml of 3-amino-9-ethyl carbazole (AEC), 0.04% H_2_O_2_, and sodium acetate buffer (50 mM, pH 5). The reaction was stopped by washing in tap water. The number of focus forming units (ffu) was determined in a Nikon TMS inverted phase-contrast microscope with a 20x objective. The virus titer was expressed as ffu/ml.

### Western blot

Cellular lysates of siRNA-transfected cells or cell lysates of untransfected WT, FcRn-KO, DPP4-KO, or double FcRn/DPP4-KO Caco-2 cells were separated by 10% SDS-PAGE; the proteins were then transferred to nitrocellulose membranes (Millipore #HATF00010). The membranes were blocked with 5% nonfat milk for 1 h at RT, followed by incubation with either rabbit anti-FcRn (Abcam #ab228975) or anti-DPP4 (Cell Signaling #67138) (dil. 1:1,000) polyclonal antibodies or with mouse anti-tubulin (Santa Cruz Biotechnology #sc-51500) monoclonal antibody for 1 h at RT. The unbound antibody was washed three times with PBS-0.1% Tween 20, and secondary goat anti-rabbit IgG or goat anti-mouse IgG peroxidase-conjugated antibodies (ThermoFisher #31460 and #31432), diluted 1:3000 in PBS-0.1% Tween 20, were added and incubated for 1 h at RT. After this time, the membranes were washed three times with PBS-0.1% Tween 20, and the peroxidase activity was detected using the SuperSignal West Pico PLUS Chemiluminescent Substrate (ThermoFisher #34580) according to the manufacturer’s instructions.

### Immunofluorescence

Cells grown on coverslips were infected with HAstV-1 at an MOI of 3. At 8 hpi, the cells were fixed with 2% paraformaldehyde in PBS for 20 min and permeabilized by incubation with 0.5% Triton X-100 in blocking buffer (1% bovine serum albumin in PBS with 50 mM NH_4_Cl) for 15 min. After washing with PBS, the coverslips were incubated overnight at 4°C with a rabbit polyclonal antibody to the HAstV-1 recombinant core capsid protein (1:2,000) [27] or with a mixture of HAstV antibody and mouse J2 monoclonal antibody (1:200) (NordicMUbio #10010200). The cells were then washed with PBS, and Alexa Fluor 488-Goat anti-rabbit IgG (ThermoFisher #A11034) and/or Alexa Fluor 568-Goat anti-mouse IgG (ThermoFisher #A11031) were added at a 1:1,000 dilution for 1 h at RT. Nuclei were stained with 30 nM DAPI (4 = ,6-diamidino-2-phenylindole) for 10 min. Coverslips were mounted on glass slides using Citifluor Mountant Media AF-1 (Ted Pella #19470), and the samples were observed under an Olympus FV1000 Confocal Upright microscope (BX61WI) with a 60x objective.

### Cell quantification

Quantification of cell nuclei was performed using the Cytation 1 Cell Imaging Multi-Mode Reader (Agilent #CYT1FAV-SN). The same slides used for confocal imaging (Fig 8A) were analyzed using Gen5 software (Agilent, version 3.14). Images were acquired with a 20x objective lens and processed using the *Image Analysis* *>* *Cellular Analysis* *>* *Cell Count* workflow with DAPI staining. Analysis parameters included an automatic threshold, object size range set between 5 µm and 100 µm, and the inclusion of primary edge nuclei and touching nuclei, which were separated using the split nuclei function. The two immunofluorescence patterns described in Fig 8A were quantified visually.

### Generation and isolation of single and double FcRn/DPP4 knockout Caco-2 cell lines

To generate single FcRn or DPP4 knockout (KO) cell lines, guide RNAs (gRNAs) were designed using the CRISPR Guide RNA Design Tool from Benchling. Two sgRNAs targeting exons 3 and 4 of the FCGRT gene, which encodes the FcRn protein, were selected (sgRNA1: 5’-CCTCCTGTACCACCTTACCG-3’ and sgRNA2: 5’-CGGTAAGGTGGTACAGGAGG-3’). For DPP4, two sgRNAs targeting exon 2 were selected (sgRNA1: 5’-CGGCTTGCAGACACCGTGGA-3’ and sgRNA2: 5’-AACCACGGGCACGGTGATGA-3’). To generate double-KO (FcRn/DPP4) cells, the FcRn-KO cell line was transfected with the DPP4-targeting sgRNAs. In all cases, the sgRNAs were cloned into the lentiCRISPRv2-Blast vector (a gift from Brett Stringer; Addgene plasmid #98293; http://n2t.net/addgene:98293; RRID: Addgene_98293) at the BsmBI site. Caco-2BBe1 cells were transfected with the final constructs using Lipofectamine 3000. Following selection with blasticidin for 2 weeks, cells were seeded into 96-well plates by limiting dilution, and single clones were screened by Western blot to confirm the absence of FcRn and DPP4 expression.

### FcRn-MAb competition ELISA

Purified virus at a concentration of 2 μg/ml in PBS (50 μl total) was incubated for 8 h at 4°C in 96-well ELISA microtiter plates. The plates were washed thrice with PBS containing 0.1% Tween 20 (PBST). The wells were blocked by adding 100 μl of 1% bovine serum albumin in PBS (BSA/PBS) and incubated at 37°C for 1 h, followed by three PBST washes. The ascitic fluid of either Nt-MAb 2D9, 3E8, 3H4, 3B4, or 3B6 [27], diluted 1:100 in BSA/PBS, was added for 1 h at 37°C. Then, a mix of MAb (1:100 in BSA/PBS) and biotinylated recombinant human FCGRT & B2M heterodimer protein (SinoBiological #CT071-H27H-B) was added at the indicated concentrations and incubated at 37°C for 1 h. The plates were washed three times with PBST and were incubated for 1h at RT with 50 μl of streptavidin conjugated to peroxidase (Zymed #43-8323) diluted 1:4000 in BSA/PBS and washed three times with PBST. The reaction was developed by adding the peroxidase substrate (TMB substrate kit, ThermoFisher #34021) according to the manufacturer’s instructions, and the optical density was measured at 450 nm (O.D. 450 nm).

### Selection of a mutant resistant to incubation with soluble FcRn

For the selection of an astrovirus mutant resistant to preincubation with recombinant soluble FcRn, we used a chimeric HAstV-1/HAstV-8 virus having the HAstV serotype 1 replication machinery and the capsid derived from a HAstV serotype 8 virus [30]. A viral lysate of this virus (titer of 6 × 10^^5^ ffu/ml) was incubated with 8.3 μg/ml of recombinant human FCGRT & B2M heterodimer protein (SinoBiological #CT009-H08H) for 1h at 37°C. The mix was then used to infect 100% confluent CaCo-2 monolayers grown in 24-well plates (seeded at 180,000 cells/well and used 48 h later) for 1h at 37°C as described above, and the unbound virus was removed by washing three times. The cell monolayers were incubated at 37°C for 24 h. Cell lysates were then prepared by three cycles of freeze-thawing. The resultant virus was then activated with 200 µg/ml of trypsin for 1 h at 37°C, and the soybean trypsin inhibitor at 200 µg/ml was added. This virus preparation was incubated again with FcRn before Caco-2 cell infection. The procedure was repeated until the FcRn-resistant phenotype was confirmed. The spike of the viruses obtained from passage 6 was sequenced as previously described [27].

### Biolayer interferometry binding assay

Biolayer interferometry data were collected using an Octet RED384 with the Data Acquisition Software (v.11.1.1.19). Binding assays were performed in a buffer containing 0.1M sodium phosphate, 150 mM NaCl, and 0.05% Tween-20 at pH 7.0. Binding assays were performed at 25^o^C with shaking at 1000 rpm. Octet HIS1K biosensors were equilibrated in the buffer for 600 seconds. Following equilibration, biosensors were loaded with the respective HAstV-8 spike protein at a concentration of 6 μg/ml for 300 seconds. Next, the biosensors were placed back into a buffer for 60 seconds to determine a baseline before being dipped into serially diluted FcRn to measure association for 120 seconds. For the WT HAstV-8 spike, the biosensors were dipped into a 5-point serial dilution consisting of 1,000 nM, 500 nM, 250 nM, 125 nM, and 62.5 nM FcRn. Due to lower affinity, mutant HAstV-8 (D471S, N512D) spike-loaded biosensors were dipped into a single FcRn concentration of 2,000 nM. After association with FcRn, the biosensors were returned to the buffer for 60 seconds to measure dissociation. Kinetics calculations were performed using the 1:1 binding model in the Octet Data Analysis HT software (v7). Each replicate was reference-subtracted with a control containing no FcRn, aligned to the baseline, and aligned to the baseline step for inter-step correction. All binding assays were run in triplicate, and the average K_D_ ± standard deviation was reported.

### RT-qPCR virus binding assay

HAstV-1 virus particles (160 ng) purified as previously described [9] were added to 100% confluent Caco-2 cell monolayers in 24-well plates (seeded at 180,000 cells/well and used 48 h later) for 1 h on ice. After this time, the unbound virus was removed by washing three times with cold PBS. Total RNA was extracted with TRIzol reagent (Invitrogen #15596018) according to the manufacturer’s instructions. cDNA was generated by reverse transcription using M-MLV Reverse Transcriptase (Invitrogen #28025013), and qPCR was performed with RealQ Plus Master Mix Green (Ampliqon #A325402) using a QuantStudio 5 Real-Time PCR (Applied Biosystems #A28134). The primers used to amplify a region of the ORF 2 of HAstV-1 were HAstV-1.1 FW 5’- GGGCATCTGGTCATGGTTAT -3’ and HAstV-1.1 RV 5’ – GTCTGTGTCTGTCTCTATGTCTTC -3’. The amount of viral RNA was normalized to that of 18S RNA using primers 18S rRNA FW, 5’- CGAAAGCATTTGCCAAGAAT -3’; 18S rRNA RV 5’- GCATCGTTTATGGTCGGAA -3’. To evaluate the binding of HAstV-1 in the presence of Nt-MAbs, the virus (160 ng) was pre-incubated for 1h at RT with a 1:100 dilution of ascitic fluid of MAb 3H4 or 3B4 to HAstV-1 [27] or MAb 2D9 to HAstV-8, which was used as a negative control. Similarly, to evaluate the binding of HAstV-1 in the presence of the soluble recombinant FcRn&B2M heterodimer (SinoBiological #CT071-H27H-B), the virus was pre-incubated with 15 or 30 µg of FcRn for 1 h at RT. Caco-2 cell monolayers grown in 24-well plates to 100% confluency (seeded at 180,000 cells/well and used 48 h later) were washed once with PBS, followed by a 15-minute incubation on ice. The cells were then washed once with ice-cold PBS and incubated with the virus-antibody complex or the virus-FcRn complex for 1h on ice, and the bound virus was determined as described above.

### Infectious virus binding kinetics assay

The binding assay was carried out as previously described [11]. Briefly, ice-cold trypsin-activated virus was added to 100% confluent Caco-2 cells (seeded at 30,000 cells/well and used 48 h later) at an MOI of 0.02. The cells were maintained in an ice-cold water bath to prevent virus entry. The unbound particles were diluted and washed out of the cells at different times. After two additional washes with ice-cold minimal essential medium (MEM) to completely remove the unbound virus, warm MEM was added, and the cells were incubated at 37°C for 18 h. Infected cells were detected by an immunocytochemistry assay as described above. Thus, infected cells represent the number of infectious particles that could bind at a given time at 4°C and subsequently enter cells at 37°C. Data were processed using the following equation: ln(V_0_/V_t_) = (KC)t, where V_0_ is the total number of input infectious particles (normalized to the number detected after 1h of adsorption at 4°C), V_t_ is the unattached virus at the indicated time (calculated as the total virus added minus the number of infected cells), C is the cell concentration, and K is the attachment rate constant. Given that the HAstV cell receptor was considered present in large excess and the virus in limited amounts, the virus-cell interaction was expected to be a first-order reaction. This assumption was confirmed by the linear graph obtained (Fig 1D); therefore, the half-time of attachment was calculated by the following equation: t_1/2 _= 0.693/k, where k is the slope of the curve.

### Infectious virus internalization assay

HAstV-1 at an MOI of 0.02 was added to 100% confluent WT or FcRn-KO Caco-2 cell monolayers in 96-well plates (seeded at 30,000 cells/well and used 48 h later) for 1h at 4ºC. The cells were switched to 37ºC and incubated for the indicated times to allow the virus to be internalized. To remove the virus adsorbed to the cell surface that had not been internalized during the incubation period at 37ºC, the cells were placed on ice and incubated with Nt-MAb 3B4 to HAstV-1 (ascitic fluid diluted 1:50; at this concentration, MAb 3B4 completely neutralizes the exposed cell surface-bound virus) for 1h at 4 ºC and then washed three times, and the infection was left to proceed for 18 h at 37ºC. The infectious, internalized virus was detected by an immunoperoxidase assay as described above.

### Virus RNA release assay

A neutral red assay was performed to determine when the virus genome is uncoated and released from the endocytic vesicle, as previously described [31]. Briefly, neutral red-labeled HAstV-1 virus stock was prepared by infecting Caco-2 cells with HAstV-1 (MOI of 0.1) in 10 µg/ml neutral red (Sigma #553-24-2) for 4 days at 37^o^C protected from light. To confirm its photosensitivity, the virus stock was titrated either in the dark or under white light. Virus infectivity was 100 times higher when the virus was kept in the dark compared to white light conditions during the infection (3.2x10^6^ vs. 3.1x10^4^ ffu/ml). To evaluate the kinetics of RNA release, two-fold dilutions of neutral red-HAstV were adsorbed to cells in 96-well plates (seeded at 30,000 cells/well and used 48 h later) for 1h at 4°C in the dark, and then the cultures were shifted to 37°C and exposed to white light at the indicated times post-adsorption; the cells were kept for 18h at 37°C, time at which the cells were fixed and the infectivity was determined as described above.

### Statistical methods

Statistical significance was evaluated by a t-test using GraphPad Prism 9.0.
